# Trichomonosis in Austrian Songbirds—Geographic Distribution, Pathological Lesions and Genetic Characterization over Nine Years

**DOI:** 10.3390/ani12101306

**Published:** 2022-05-19

**Authors:** René Brunthaler, Norbert Teufelbauer, Benjamin Seaman, Nora Nedorost, Karin Bittermann, Julia Matt, Christiane Weissenbacher-Lang, Herbert Weissenböck

**Affiliations:** 1 Department for Pathobiology, Institute of Pathology, University of Veterinary Medicine Vienna, Veterinärplatz 1, 1210 Vienna, Austria; rene.brunthaler@vetmeduni.ac.at (R.B.); nora.nedorost@vetmeduni.ac.at (N.N.); karin.bittermann@vetmeduni.ac.at (K.B.); julia.matt@vetmeduni.ac.at (J.M.); christiane.weissenbacher-lang@vetmeduni.ac.at (C.W.-L.); 2 BirdLife Austria, Museumsplatz 1/10/8, 1070 Vienna, Austria; norbert.teufelbauer@birdlife.at (N.T.); benjamin.seaman@birdlife.at (B.S.)

**Keywords:** *Trichomonas gallinae*, trichomonosis, Austria, greenfinch, in situ hybridization, genetic characterization

## Abstract

**Simple Summary:**

There are many causes of mortality in free-living songbirds, and, usually, the general public is not particularly interested in these issues. Only when large numbers of dead birds are observed within small geographic areas and within a short period of time is some public attention focused on these phenomena. In this study, we investigated episodes of mass mortality of songbirds, especially in greenfinches all over Austria, which occurred between 2012 and 2020. We noticed that all of these losses were caused by a particular protozoal parasite (*Trichomonas* (*T.*) *gallinae*) which induced severe inflammation of the upper digestive tract and subsequently death due to starvation. We were able to establish clear links with almost identical disease outbreaks in other countries and thus trace the path of the pathogen through Europe. It could be shown that *T. gallinae* has spread over the whole of Austria in recent years and occurred in many different songbird species, with a preference for greenfinches, leading to a significant decline in the population of this bird species. Since most birds are presumably infected at feeding sites, hygiene conditions and the correct selection of feeders play an important role in the prevention of disease.

**Abstract:**

In the early summer of 2012, sudden mass mortality among songbirds, particularly in greenfinches (*Chloris chloris*, syn: *Carduelis chloris*) was observed in Austria, which was caused by the protozoan parasite *Trichomonas gallinae*. This pathogen induced fibrinonecrotic ingluvitis and/or esophagitis, leading to impairment of food intake and ultimately death due to starvation. The pathogen was successfully detected within the lesions by polymerase chain reaction (PCR) and chromogenic in situ hybridization. The epizootic resulted in a significant decline in the Austrian greenfinch population. Continuing passive surveillance in the subsequent years (2013–2020) revealed that the condition occurred each year and was present in the entire country. Genetic characterization of the pathogen showed the presence of an identical strain irrespective of geographical location, bird species, and year.

## 1. Introduction

Greenfinches (*Chloris chloris*, syn.: *Carduelis chloris*) were among the 15 most common species of songbirds in Austria [1]. In 2012, the conservation organization BirdLife Austria estimated a nationwide population of around 235,000 breeding pairs. In the last years, however, this population has decreased by more than 50%. Mass mortality of wild Passeriformes is not an unusual event in wildlife, and, often, infectious diseases are considered to be the main cause [2]. A massive population decline in greenfinches was observed in parts of Europe and has been associated with outbreaks of infections with the protozoan parasite *Trichomonas* (*T.*) *gallinae* [3,4,5,6]. The highly infectious trichomonads are usually taken up via drinking water, where they can survive for about 24 h. Further sources of infection may be partner feeding in old birds or feeding of young animals in the nest. The infection chain is further maintained by small numbers of parasites, which may reside in the crop of subclinically infected reservoir birds [7]. Immune suppressive events, such as stress situations such as low food availability, anthropogenic pressures such as changes in land use and pollution, or other concurring diseases, may be a trigger of increased mortality rates in infected birds [7]. Mass deaths of passerine birds, especially finches, due to trichomonosis, sometimes in association with a marked decline in population, have already been observed in the British Isles, Canadian Maritime provinces, Southern Fennoscandia (Norway, Sweden, Finland), Germany, The Netherlands, France, and Slovenia [5,8,9,10,11,12,13,14,15,16,17,18]. In all these reports, as well as in our present study, macroscopic or histological findings provided no evidence for any other additional infectious causes of the high mortality rate. 

A typical sign of infestation with trichomonads is vomiting of feed mixed with sticky mucus. Some birds do not eject mucus or feed but only perform “dry” chewing movements. Frequent sneezing may also be observed. In addition, due to the accumulation of fibrinonecrotic masses in the mucosa, severe swelling of the crop may occur. Some birds are also dyspneic. From the beak cavity, they may emit a foul to fishy smell. Generally, diseased birds are dull, apathetic, and show a ruffled plumage. Due to the nodular masses, which may entirely obstruct the lumen of the upper digestive tract, feed intake is severely compromised, and the birds subsequently lose weight and finally die. 

The flagellated protozoan parasites colonize the mucous membranes of the pharynx, crop, and esophagus, where they induce a fibrinonecrotic inflammation. In very severe cases, the inflammation invades the mandibular bone or causes severe periesophageal fasciitis, cellulitis, and/or myositis [14]. Another site of inflammation associated with the presence of *T. gallinae* in finch birds is the eyelid [19]. 

In this study, we report on the first severe outbreak with mass mortality of greenfinches and other wild songbird species in Austria due to trichomonosis and the further course of this disease over the subsequent eight years. The emphasis was placed on the geographic location of mortality events, associated pathological changes, diagnostic investigations, and genetic characterization of the causative parasite strain.

## 2. Materials and Methods

### 2.1. Sample Collection and Laboratory Investigations

In the early summer months (May, June, and July) of 2012, an episode of mass mortality among songbirds in different parts of Austria was registered. These observations were made by the attentive general public as well as by ornithologists. The dead animals were mainly found near feeding sites. The observers also noticed obviously weak birds sitting on the ground and not flying away when approached. The carcasses were sent to the Institute of Pathology of the University of Veterinary Medicine in Vienna. The animals were subjected to a standardized examination procedure. First, species, age, sex, weight, and exact location of origin were recorded. Subsequently, as far as the state of preservation allowed, necropsy was performed, and tissue samples of heart, lung, liver, spleen, kidneys, crop, proventriculus, stomach, and intestine were taken for histological examination. The samples were fixed in 4% buffered formaldehyde solution (10% formalin), dehydrated in graded series of ethanol, and embedded in paraffin wax. Thereafter, approximately 3 μm thick sections were stained with hematoxylin and eosin (H&E). Additional sections were also subjected to Grocott’s methenamine silver nitrate (GMS) staining of the digestive tract when there was histologic evidence of additional fungal disease. Selective isolation of *Salmonella* spp. was attempted from the crop lesions of the first four dead birds submitted in 2012. In all animals, a previously described PCR assay for the detection of trichomonads [20], and in all animals, a previously described chromogenic in situ hybridization (ISH) [21] was carried out. After the mass die-off of songbirds in 2012, increased attention was paid to songbird mortality events, and over the next eight years (2013–2020), dead birds were repeatedly collected, examined, and subjected to the investigations already described above. 

### 2.2. DNA Extraction for Sequence Analysis

DNA was extracted from 31 native crop tissue samples and 3 pooled samples, each consisting of lung and liver tissue, using the Nexttec clean column kit (Nexttec^TM^ Biotechnologie GmbH, Hilgertshausen, Germany) according to the manufacturer’s instructions.

### 2.3. Trichomonad Partial Genome Sequencing

The genetic heterogeneity of the trichomonads present in the birds was investigated based on the partial sequence identity of the ITS1-5.8S-ITS2 region [15,22] and the Fe-hydrogenase gene [23]. Both protocols were modified. The PCR reaction master mixture consisted of 12.5 µL of KAPA2G Fast HotStart ready mix with dye (Sigma-Aldrich, Vienna, Austria), 0.4 µM of each primer, 2 µL of template DNA, and distilled water to a total volume of 25 µL per reaction. The cycling parameters consisted of an initial denaturation at 95 °C for 3 min, followed by 40 cycles at 95 °C for 15 s, 55 °C for 15 s, and 72 °C for 25 s with a final extension step at 72 °C for 1 min. The successful amplification was checked by gel electrophoresis by analyzing an aliquot of 10 μL of each PCR product on a 2% Tris acetate–EDTA–agarose gel. The agarose gel was stained (ROTI^®^ GelStain; Lactan, Graz, Austria), and bands were detected (Molecular Imager, GEL DOC^TM^ XR+, BioRad Laboratories, Vienna, Austria). PCR products of the expected sizes (ITS1-5,8S-ITS2 region: 526 bp, Fe-hydrogenase gene: 993 bp) were submitted for Sanger DNA sequencing (Microsynth, Vienna, Austria). The obtained nucleotide sequences were assembled using the software BioEdit Sequence Alignment Editor 7.0.5.3 and analyzed using a BLAST search of the NCBI GenBank database [24].

### 2.4. Bird Monitoring Counts

The Austrian “Brutvogel-Monitoring” is a monitoring scheme of common breeding birds coordinated by BirdLife Austria since 1998 [25]. The counts are conducted by experienced volunteers throughout the country twice annually during the breeding season. Each monitoring “site” (comprised of approx. 10–15 count points) is visited by the same volunteers every year, at the same time of day, and using the same survey method. Volunteers spend exactly five minutes at each count point and register the number of individuals of each species detected. Since the start of the program in 1998, the number of monitoring sites has increased from approx. 150 to almost 300 in 2020. Annual numbers are pooled for each site using the maximum number of individuals registered at each count point during that year’s two survey periods. Population trends are then calculated using log-linear Poisson regressions [26]. The first year of the program is set as the 100%-mark, and a population index value with corresponding confidence intervals is calculated for every year since then. The width of the confidence intervals is largely dependent on sample size, which has improved for most species with the increasing number of monitoring sites.

## 3. Results

### 3.1. Dead Bird Reports

**Figure 1:**
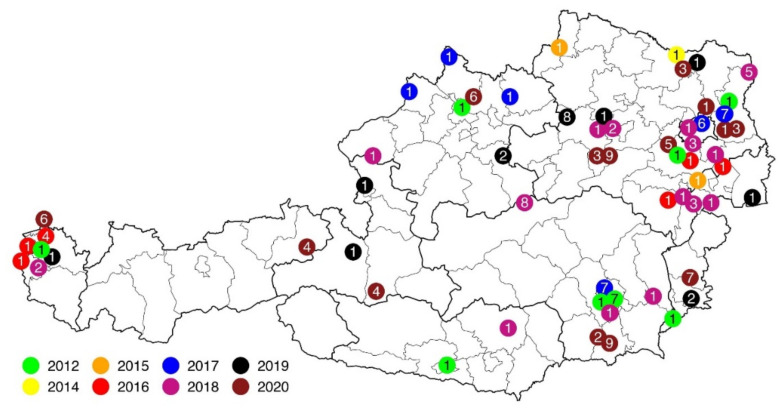
Map of Austria showing locations of detected cases of trichomonosis in songbirds from 2012 to 2020. Each color represents a particular year; the number in the circle determines the bird species: (1) Greenfinch; (2) Chaffinch; (3) Goldfinch; (4) Bullfinch; (5) Hawfinch; (6) Eurasian siskin; (7) Great tit; (8) Yellowhammer; (9) Brambling.

During the first epidemic in the summer of 2012, 16 entire animal carcasses, as well as one tissue sample of the crop of a deceased bird, were submitted by private individuals or via BirdLife Austria for clarification of the cause of death. Most of these dead songbirds had been found in the immediate vicinity of feeders or at places where drinking water was offered. Attentive birdwatchers reported dozens of dead birds or birds that were unable to fly away, showing markedly fluffed plumage and beak cavities appearing to be congested with food remains. The places where dead birds were found were distributed over several provinces of Austria (Vorarlberg, Upper- and Lower Austria, Carinthia, Burgenland, and Styria) (Figure 1). The majority (15) of the birds were greenfinches, and two birds were great tits (*Parus major*) (Table S1). All were adults, of which eight greenfinches could be identified as female and four as male. In the remaining five birds, the sex could not be determined.

In 2013, no exceptionally increased mortality in the wild-living songbird population of Austria was recorded, and no songbirds were submitted for necropsy. Between 2014 and 2020, 71 songbirds were submitted to determine the cause of disease or death. These birds were rarely in an excellent or good preservation status; some had been dead for several days or had been frozen after collection. Again, the majority of submitted birds were greenfinches, with 44 individuals. Furthermore, carcasses of six chaffinches (*Fringilla coelebs*), five goldfinches (*Carduelis carduelis*), three bullfinches (*Pyrrhula pyrrhula*), three great tits, three hawfinches (*Coccothraustes coccothraustes*), three Eurasian siskins (*Spinus spinus*, syn.: *Carduelis spinus*), two bramblings (*Fringilla montifringilla*) and two yellowhammers (*Emberiza citrinella*) were received (Table S1). These wild birds had also been found in sick or moribund conditions, mainly in gardens at year-round feeding sites. Over the years from 2014 to 2020, increased deaths of finch birds were observed in all nine federal states of Austria. All were adults, of which 28 greenfinches, two chaffinches, four goldfinches, one bullfinch, two siskins and two yellowhammers were male, and three greenfinches, one great tit, and one hawfinch were female. In the remaining 27 birds, the sex could not be determined. 

### 3.2. Macroscopic Findings

**Figure 2:**
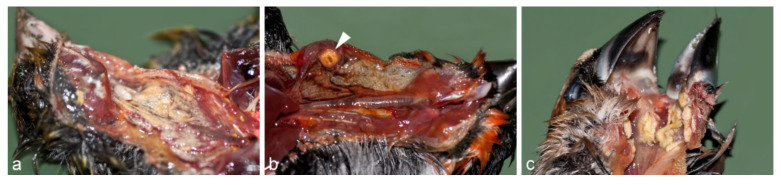
(**a**) Diphtheritic pseudomembrane affecting the esophagus, crop and pharynx of a greenfinch as multifocal to coalescing nodules in the crop obstructing the lumen; (**b**) Focal diphtheritic pseudomembrane of a bullfinch as a single nodule in the crop mucosa; (**c**) Hawfinch with extensive diphtheritic pseudomembrane affecting the pharynx.

The most striking change at macroscopic examination was a very bad nutritional condition with considerable atrophy of the pectoral muscles and complete loss of fat reserves. The average body weight of the greenfinches was 19.2 g (compared to a physiological weight of 24–30 g [27]. Similarly, the majority of the other songbird species showed signs of emaciation with moderate to severe atrophy of the pectoral muscles. Only four chaffinches, one goldfinch, and one bullfinch had a physiological weight. In 84 birds, macroscopically, either single nodular, multifocal to coalescing, or diffuse yellowish diphtheritic pseudomembranes of the mucosa were found in the beak cavity, pharynx, cranial part of the esophagus, and crop (Figure 2). The submitted crop sample showed the same changes. Due to these protruding lesions of the mucosa, the lumen was considerably narrowed or even completely obstructed. In most cases, the proventriculus and gizzard were nearly empty, and large amounts of blood were seen in the intestinal lumen of at least 23 birds. No other pathomorphological abnormalities occurred. 

### 3.3. Histological Findings, PCR, In Situ Hybridization and Bacteriological Examination

**Figure 3:**
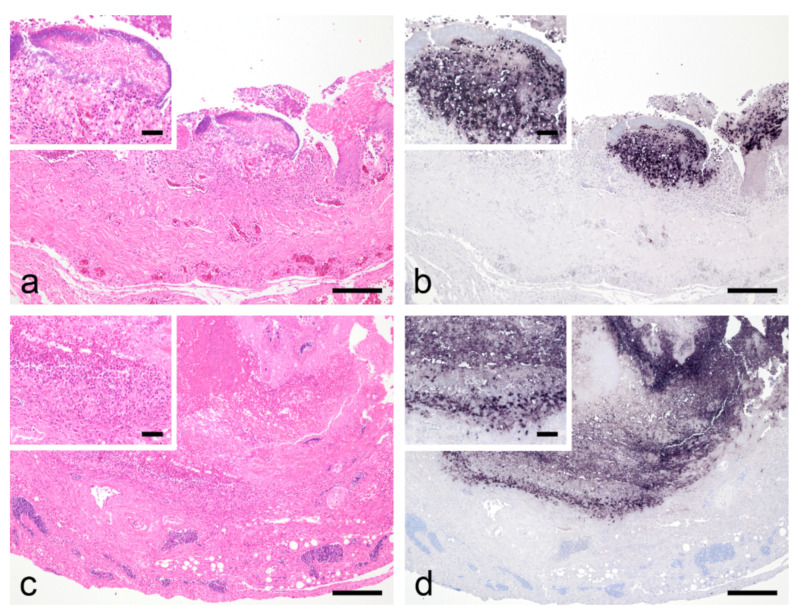
(**a**) Greenfinch, esophagus with predominantly superficial heterophilic inflammation, mucosal ulceration and bacterial growth (H&E staining, bar = 160). Insert shows fibrin formation, infiltration of heterophils, intralesional and superficial coccoid bacteria (H&E staining, bar = 40 µm); (**b**) Esophageal wall of the same bird; ISH reveals abundant colonization with *T. gallinae* appearing as purple to black stained objects (bar = 160). Insert shows higher magnification of positive ISH (bar = 40 µm); (**c**) Bullfinch, esophagus with severe transmural heterophilic inflammation and necrosis (H&E staining, bar = 160). Insert shows high-grade diffuse infiltration with heterophils (bar = 40 µm); (**d**) Esophageal wall of the same bird; ISH reveals large amounts of purple to black stained protozoal objects corresponding to *T. gallinae* within the necrotic and inflamed tissue sites (bar = 160). Insert shows higher magnification of positive ISH (bar = 40 µm).

Histologically, there was a severe acute, frequently transmural fibrinoheterophilic to necrotizing pharyngitis, ingluvitis, or esophagitis with abundant superficial predominantly coccoid bacterial colonies (Figure 3). Salmonella spp. were not found by bacteriological examination in the first four dead birds. However, ISH with a probe directed against the majority of representatives of the order Trichomonadida localized abundant specific signals in all studied pharynx, crop, or esophagus lesions (Figure 3). PCR for the detection of trichomonads also yielded a positive result in the examined tissue samples. In four birds, small numbers of *Macrorhabdus ornithogaster* were identified on the mucosal surface at the transition of the proventriculus to gizzard by H&E and GMS staining. There were no associated inflammatory reactions. No other relevant pathologic lesions occurred.

Of the 88 birds examined, only one greenfinch, one great tit, and one Eurasian siskin showed neither macroscopically nor histologically detectable lesions. However, Trichomonas spp. were also detected in the crop tissue of these animals by PCR. 

### 3.4. Sequence Analysis

Thirty-three samples from 2017 to 2020 could be sequenced successfully on the ITS1-5.8S-ITS2 region (NCBI GenBank acc. no. OL678475-OL678507) and 25 on the Fe-hydrogenase gene (NCBI GenBank acc. no. OL654280-OL654304) (Table S1). All acquired sequences were identical and showed 100% identity with published *T. gallinae* sequences (GenBank acc. no. ITS1-5.8S-ITS2 region: MN385401, Fe- hydrogenase gene: MK172852).

### 3.5. Greenfinch Monitoring Data

**Figure 4:**
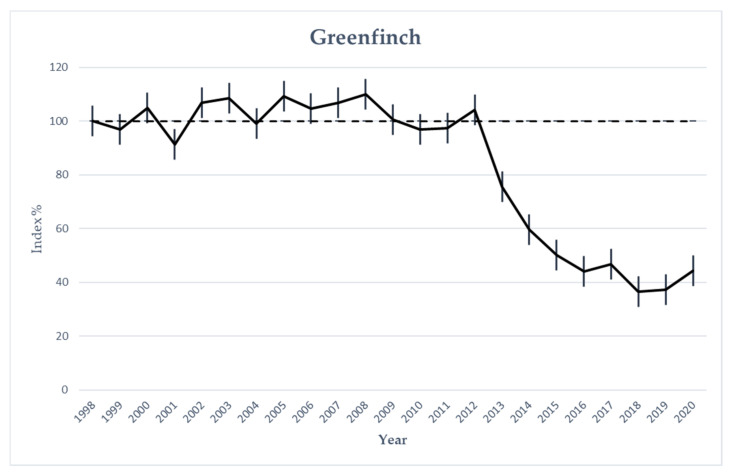
Graphical representation of population trend (index values and 95%-confidence limits) of the Austrian greenfinch population for the period 1998–2020 (1998 = 100%).

Since the mass mortality of wild songbirds in 2012, a striking gradual decline in Austria’s greenfinch population has been observed. BirdLife Austria’s annual breeding bird monitoring counts showed a reduction of up to 60% of this bird species between 2012 and 2020 (Figure 4).

## 4. Discussion

The unicellular flagellate *T. gallinae* is the causative agent of avian trichomonosis, also known as canker in pigeons or as frounce in falcons. This pathogen occurs worldwide and can be found in many other bird species belonging to the orders of Anseriformes [28], Galliformes [29,30], Gruiformes [31], Passeriformes [31,32], Piciformes [33], Psittaciformes [34,35] and Struthioniformes [28]. In general, the typical lesions occur in the beak cavity, infraorbital sinus, pharynx, esophagus, crop, and also in the proventriculus. In addition, the liver or heart and, less frequently, other internal organs may show changes, which usually appear macroscopically as yellowish, cheesy necrotic lesions [36]. Pigeons are very frequently affected, as documented by reports on *T. gallinae*-associated mass mortalities in free-living mourning doves *(Zenaida macroura)* in Florida and Alabama, wood pigeons *(Columba palumbus)* in Spain and Portugal, and band-tailed pigeons *(Patagioenas fasciata)* in California [37,38,39,40].

In finches, symptomatic trichomonosis was initially only known in experimentally infected, immunosuppressed birds [41]. However, mass mortalities due to natural infections with *T. gallinae* were described for the first time in the greenfinch population in the UK in 2005 [4,9]. In the following years, a successive spread of trichomonads was detected in finch populations in continental Europe and westward to Canada, with episodes of increased mortality in free-ranging songbirds in Wales, Ireland, southern Scandinavia, the Netherlands, France, Germany, and finally in Austria and Slovenia in 2012 [4,5,14,15,16,17,18,42]. During the first epidemic in the summer of 2012, dead songbirds, in particular, greenfinches, were spotted over almost the entire Austrian territory. After no trichomonads-associated deaths in the finch population were reported to us in Austria in 2013, there was a steady increase in finch trichomonosis from 2014 onwards, with a peak in 2018 and 2020. Interestingly, over the years, the number of bird species found to be infected with *T. gallinae* increased. Still, greenfinches remained the most affected bird species, but other Fringillidae, such as chaffinches, goldfinches, bullfinches, hawfinches, Eurasian siskins, and bramblings, and also songbirds belonging to the families *Paridae* (great tits) or *Emberizidae* (yellowhammers) succumbed to the disease. The large majority, however, were finches. Thus, our observations are consistent with those from other studies indicating the emergence and spread of trichomonosis, particularly in the finch population [3,4,5,6,9,14,15,16,17,18,42,43]. Trichomonosis in passerine birds was unknown in Austria before 2012, and this raises the question of how the pathogen actually was introduced to this country. According to Lawson et al., the most likely reason for the quite rapid transboundary spread of finch trichomonosis in Europe might have been seasonally migrating chaffinches [43], which use flyways directly from England across the North Sea to southern Scandinavia in spring and migrate back in autumn across the English Channel via Denmark, Germany, the Netherlands and Belgium [44]. This is consistent with the timing of the occurrence of finch trichomonosis in Germany and the Netherlands in 2009 and in France in 2010 [5,15,18]. The greenfinch is native to the entire European continent and is actually an annual bird, but migrations to western and south-western Europe have been recorded [45]. Thus, greenfinches breeding in Fennoscandia use overwintering sites in Belgium, The Netherlands, and Luxembourg [46]. This might relate to the spread of the disease southwards to Austria and Slovenia, which could be linked to less well-documented migration routes of finches. The vast majority of affected birds showed fibrinopurulent to fibrinonecrotizing pharyngitis, ingluvitis, or esophagitis with extensive superficial bacterial growth, which may have had a negative influence on the course of the disease due to possible acute to peracute septicemia. The inflammatory lesions presented as multifocal to confluent yellow nodules or as diffuse, yellowish, longitudinal thickenings of the mucosa of the pharynx, crop, and esophagus. As these lesions sometimes significantly obstructed the lumen of the upper digestive tract and the inflammation of the mucous membranes most likely caused severe pain, the birds’ feed intake was considerably impeded. In accordance with the cases reported from Canada and southern Fennoscandia [13,14], the vast majority of finches in our study showed marked emaciation with atrophy of the pectoral muscles and sometimes complete loss of fat reserves. In budgerigars (*Melopsittacus undulatus*), weight loss and subsequent death may occur due to constant vomiting and regurgitation as well as anorexia [34]. In finches that died of trichomonosis in Austria, findings suggesting regurgitation were also found. The beaks were regularly congested with feed, and frequently also, the pharynx was filled with grain material. According to Kocan et al., infection with *T. gallinae* can cause lesions large enough to obstruct the esophagus and thus lead to starvation [47]. In addition, the proventriculus and gizzard were often empty, and large amounts of blood could be found in the intestinal tract, suggesting a hemorrhagic diathesis in the intestine, which is indicative of approximately 24 to 36 h of food abstinence [48]. In the case of fibrinopurulent inflammation of the pharyngeal, crop, and esophageal mucosa, infection with *Salmonella typhimurium*, yeasts or poxviruses must be considered in addition to trichomonosis [5]. In particular, infections with *Salmonella* spp. in passerine birds are responsible for frequent deaths with almost identical pathomorphological changes of the upper digestive tract with yellowish nodular enlargements in the mucous membranes [49,50,51,52,53,54,55,56,57,58,59,60,61,62,63,64]. Since the lesions in the pharynx, crop, and esophagus are morphologically indistinguishable from those of a trichomonad infection, further etiological investigations must be carried out for differential diagnosis. Microscopic examination of the throat and crop swabs from living or freshly dead birds is well suited for the diagnosis of trichomonads [65,66]. The problem, however, is that these pathogens are extremely sensitive to environmental conditions and require a warm, moist environment to survive but do not form true cysts [16,67]. It is thought that *T. gallinae* may have a transient cyst-like stage (pseudocyst) and, therefore, may increase the likelihood of out-of-host survival [68]. For example, Neimanis et al. were able to detect viable parasites in only 22 to 38% of samples from autolytic birds in their study. Therefore, it can be stated that the diagnosis of a trichomonad infection by postmortal microscopic examination of swabs is not achieved in all cases since bird carcasses are often received with considerable autolytic changes or after being stored in a freezer for some time. According to our experience, trichomonads are also difficult to detect by light microscopic investigation of tissue sections. Frequently, the affected tissues are so severely necrotic that in conventional H&E stained slides, unequivocal identification of the protozoa is difficult or impossible [14]. Therefore, we relied on ISH for the visualization of the trichomonads. This method not only allowed the localization of the protozoal pathogens directly in the tissue but also to correlate them with the accompanying lesions and estimate the parasitic load, which enabled an assessment of pathogenicity [69]. ISH shows robust, specific labeling of protozoal RNA even in necrotic, autolyzed, or frozen tissue. Another reliable diagnostic method for the diagnosis of trichomonad infections in autolytic and frozen material or after formalin fixation and embedding in paraffin wax was a PCR previously developed for this purpose by our group [20]. The PCR protocols used for the generation of amplification products for subsequent sequencing were less successful because the insufficient preservation conditions of the samples obviously led to DNA fragmentation, not allowing for amplification of the nearly 1000 bp fragment in case of the Fe-hydrogenase gene in some of the samples. In cases of successful amplification and sequencing of the ITS1-5.8S-ITS2 region and the Fe-hydrogenase gene, a 100% sequence identity among all Austrian samples investigated between 2012 and 2020 in our study and by Ganas et al. could be demonstrated [42]. Furthermore, our sequences were 100% identical with sequences derived from trichomonads of Dutch, French and Canadian finches on both genetic loci [15,16,70] and of southern Scandinavian and British finches on the ITS1-5.8S-ITS2 gene locus [6,43]. Our results, therefore, support the findings of Lawson et al. that a single genetically stable clonal strain of *T. gallinae* is responsible for trichomonosis in finch birds in Europe and also in the Canadian maritime provinces [23]. 

A major epidemiological factor for the perpetuation of the epidemic is certainly the feeding of songbirds in private gardens throughout the year, where water bowls or water baths are provided [6,14,71,72]. Although the parasite can only survive in the environment for a short period of time, these artificial devices are considered potential sources of infection as water or feed can easily become contaminated with saliva, vomit, or feces [34,47]. The estimated maximum survival time of trichomonads in water is 26 h [73]. According to Zentralanstalt für Meteorologie und Geodynamik (ZAMG), 2012 was one of the warmest summers in the history of measurements in Austria [74], so many different bird species probably increasingly visited water points near feeding areas. Finches tend to stay in feeding areas for prolonged periods of time and eat on the ground. As a result, these birds are more likely to defecate and come in contact with contaminated feed [55]. In contrast, other bird species, e.g., black-capped chickadees (*Poecile atricapillus*), fly to the feeders only very briefly to grab individual seeds [55]. The time they spend there is, therefore, much shorter, and the risk of infection is lower [55]. According to Refsum et al., finches are very gregarious and prefer to feed in larger groups on the ground, which easily results in infections via contaminated seeds [57]. Greenfinches, in particular, often feed each other grains from their beaks [5]. In addition, finches usually hold several grains in their beaks and peel them one after the other [57]. This could be another factor for the increased incidence of the disease in greenfinches. 

As mentioned above, *T. gallinae* is also found worldwide in other bird species. Columbiformes, and particularly *Columba livia* are considered primary reservoirs of this protozoan pathogen, with broods usually being infected with these flagellates via crop milk after hatching or transmission taking place between mating partners via the oral route [75,76,77]. Interestingly, in the ITS1-5.8S-ITS2 region, there is 100% sequence homology of the *T. gallinae* strains detected in the songbirds of our study with strains from rock pigeons (*Columba livia*) from Spain, China, and in several dove species from different regions of western and southern Europe [78,79,80]. Ganas et al. also detected the same strain in a feral pigeon (*Columba livia domestica)* from Austria. According to Marx et al., turtle doves *(Streptopelia turtur)*, collared doves *(Streptopelia decaocto)*, stock doves (*Columba oenas),* and wood pigeons (*Columba palumbus)* in particular have a high prevalence of *T. gallinae*. Similar results were found in the United Kingdom, where *Trichomonas* was detected in adult wood pigeons, collared doves, stock doves, turtle doves, and some of their nestlings from Essex, Norfolk, and Suffolk [81]. When comparing these sequences, especially from turtle doves, with our sequences from Austrian songbirds, a 100% match in the ITS1-5.8S-ITS2 region is found. These data support the assumption that transmission of trichomonads between pigeons and finches occurs under natural conditions, probably at jointly used feeding and watering places.

In Slovenia, a *T. gallinae*-associated disease with several deaths occurred in a flock of canaries [82]. It is very likely that this event was directly related to the finch trichomonosis already circulating in Slovenia at that time because the acquired sequences show 100% identity to the strains of a free-living greenfinch and are also completely identical to the sequences in our study [42]. 

Birds of prey can also contract trichomonosis in case their prey [71,75,76,78,83,84,85,86,87], pigeons or small songbirds, are infected with *T. gallinae,* and they develop granulomas in the beak cavity and pharyngeal mucosa [76,78]. Interestingly, sequences of trichomonads infecting a barn owl (*Tyto alba)*, a Bonelli’s eagle (*Aquila fasciata*) and a kestrel (*Falco tinnunculus)* from the Valencia area, and sparrow hawks (*Accipiter nisus)* from the Czech Republic showed complete identity in the ITS1-5.8S-ITS2 with sequences of Austrian finch trichomonosis cases [78,88]. In the course of our investigations, however, we did not become aware of increased trichomonad infections in other bird species, except songbirds, in Austria. 

One clear commonality recorded by all authors is the time of year when deaths due to finch trichomonosis occur. *T. gallinae* infections were predominantly observed in the warmer season, from spring to early autumn [3,4,6,13,14,15,16,17,18]. In the vast majority of cases, our observations confirmed these findings. Only occasionally did *T. gallinae*-related deaths occur in winter. This can be explained by the dependency of trichomonads on a warm, humid environment and their sensitivity to environmental conditions [67]. In contrast, morphologically indistinguishable inflammations of the crop mucosa due to *Salmonella typhimurium* mainly occur in the winter months [64].

Co-infections with other pathogens as possible predisposing factors for the widespread emergence of trichomonosis have not been described previously and seem to have not been in the focus of former reports. Furthermore, the present work does not contribute much to this issue. We only were able to exclude *Salmonella* in a part of the samples and to confirm *M. ornithogaster* in several investigated birds. In a separate publication by our group, frequent co-infections with haemosporidian parasites were reported in cases with finch trichomonosis [89].

Robinson et al. and Lehikoinen et al. described a significant decline in the finch populations of the United Kingdom and Finland, especially in greenfinches and chaffinches [3,6]. Both studies suggest that finch trichomonosis is largely responsible for this decline in bird numbers. The authors of a Dutch study also assume that trichomonosis is similarly lethal in their country as in the UK, but the impact on the greenfinch population in the available bird census data was probably not yet conclusive enough [15]. However, a continuous decline, especially in the greenfinch population, has also been observed in Austria over the last nine years, which is probably closely related to cases of trichomonosis. Data on the development of the Chaffinch population in Austria showed a moderate decline of approximately 11% since 1998, though the majority of this decline (8%) occurred between 2019 and 2020, the year with the last available data [BirdLife Austria, unpublished].

## 5. Conclusions

Our investigations showed that a single genetically stable clonal strain of *T. gallinae* was responsible for increased mortality in Austria’s free-living finches between 2012 and 2020 and was also a significant cause of the decline in the greenfinch population. Furthermore, with the help of molecular investigations, it was possible to demonstrate that there is a high probability of a direct connection with the mass deaths of finches in other parts of Europe and that trichomonosis has spread strongly in the wild songbird population of Austria in recent years.

## Data Availability

The data will be available with the corresponding author upon request.

## Supplementary Materials

The following supporting information can be downloaded at: https://www.mdpi.com/article/10.3390/ani12101306/s1, Table S1: Postmortem findings and results of laboratory examinations of 88 adult wild birds of the order Passeriformes, from 2012 to 2020 in Austria.
