# Supplementary material for: Trichomonosis in Austrian Songbirds—Geographic Distribution, Pathological Lesions and Genetic Characterization over Nine Years
Source: Animals (Basel). 2022 May 19;12(10):1306. doi: 10.3390/ani12101306 (PMC9137778; doi:10.3390/ani12101306)
Supplement: Supplementary file 1 [file animals-12-01306-s001.zip › animals-1713672-supplementary.pdf]

Table S1. Postmortem findings and results of laboratory examinations of 88 adult wild birds of the order Passeriformes, from 2012 to 2020 in Austria.

								GenBank accession numbers	
Species	Location	Date found	Sex/ Age	Body Condition	Weight (g)	Crop lesions	Detection method	ITS1-5.8S-ITS2	Fe-Hydrogenase-gene
Greenfinch	Hartkirchen (UA)	10.05.2012	F/A	emaciated	20	+	ISH/PCR	ND	ND
Greenfinch	Hartkirchen (UA)	10.05.2012	M/A	emaciated	18	+	ISH/PCR	ND	ND
Greenfinch	Hartkirchen (UA)	10.05.2012	F/A	emaciated	18	+	ISH/PCR	ND	ND
Greenfinch	Hartkirchen (UA)	10.05.2012	F/A	emaciated	20	+	ISH/PCR	ND	ND
Greenfinch	Graz (St)	16.05.2012	x	emaciated	16	+	ISH/PCR	ND	ND
Greenfinch	Graz (St)	16.05.2012	x	emaciated	18	+	ISH/PCR	ND	ND
Great tit	Graz (St)	16.05.2012	x	moderate	16	+	ISH/PCR	ND	ND
Great tit	Graz (St)	16.05.2012	x	moderate	16	+	ISH/PCR	ND	ND
Greenfinch	Jennersdorf (B)	01.06.2012	M/A	emaciated	22	+	ISH/PCR	Ganas et al. (2014)	
Greenfinch	Matzen (LA)	01.06.2012	F/A	emaciated	22	+	ISH/PCR	ND	ND
Greenfinch	Matzen (LA)	01.06.2012	F/A	emaciated	18	+	ISH/PCR	ND	ND
Greenfinch	Dornbirn (V)	28.06.2012	M/A	emaciated	16	+	ISH/PCR	ND	ND
Greenfinch	Villach (C)	28.06.2012	x	x	x	+	ISH/PCR	ND	ND
Greenfinch	Siegenfeld (LA)	25.07.2012	F/A	emaciated	18	+	ISH/PCR	Ganas et al. (2014)	
Greenfinch	Siegenfeld (LA)	25.07.2012	F/A	emaciated	20	+	ISH/PCR	ND	ND
Greenfinch	Siegenfeld (LA)	25.07.2012	M/A	emaciated	20	+	ISH/PCR	ND	ND
Greenfinch	Siegenfeld (LA)	25.07.2012	F/A	emaciated	22	+	ISH/PCR	ND	ND
Greenfinch	Seefeld-Kadolz (LA)	30.05.2014	M/A	emaciated	16	+	ISH/PCR	ND	ND
Greenfinch	Seefeld-Kadolz (LA)	30.05.2014	M/A	emaciated	18	+	ISH/PCR	ND	ND
Greenfinch	Seefeld-Kadolz (LA)	30.05.2014	M/A	emaciated	18	+	ISH/PCR	ND	ND
Greenfinch	Seefeld-Kadolz (LA)	30.05.2014	M/A	emaciated	22	+	ISH/PCR	ND	ND
Greenfinch	Seefeld-Kadolz (LA)	30.05.2014	M/A	emaciated	16	+	ISH/PCR	ND	ND
Greenfinch	Seefeld-Kadolz (LA)	30.05.2014	M/A	emaciated	20	+	ISH/PCR	ND	ND
Greenfinch	Neufeld/L (LA)	03.08.2015	F/A	emaciated	18	+	ISH/PCR	ND	ND
Greenfinch	Gmünd (LA)	14.08.2015	F/A	emaciated	20	+	ISH/PCR	ND	ND
Bullfinch	Dornbirn (V)	22.06.2016	x	emaciated	20	+	ISH/PCR	ND	ND
Greenfinch	Sommerein (LA)	04.07.2016	M/A	emaciated	24	+	ISH/PCR	ND	ND
Greenfinch	Neunkirchen (LA)	01.09.2016	M/A	x	x	+	ISH/PCR	ND	ND
Greenfinch	Baden (LA)	25.10.2016	M/A	emaciated	20	+	ISH/PCR	ND	ND
Greenfinch	Lustenau (V)	25.10.2016	M/A	emaciated	18	+	ISH/PCR	ND	ND
Greenfinch	Lustenau (V)	25.10.2016	M/A	emaciated	25	+	ISH/PCR	ND	ND
Greenfinch	Götzis (V)	25.10.2016	M/A	emaciated	16	+	ISH/PCR	ND	ND
Greenfinch	Lustenau (V)	25.10.2016	M/A	emaciated	20	+	ISH/PCR	ND	ND

Species	Location	Date found	Sex/ Age	Body Condition	Weight (g)	Crop lesions	Detection method	GenBank accession numbers ITS1-5.8S-ITS2	Fe-Hydrogenase-gene
Greenfinch	Brunnenthal (UA)	12.07.2017	x	emaciated	20	+	ISH/PCR	ND	ND
Greenfinch	Neumarkt (UA)	21.07.2017	x	x	x	0	PCR	ND	ND
Greenfinch	Neumarkt (UA)	21.07.2017	x	x	x	+	ISH/PCR	OL678475	OL654280
Greenfinch	Neumarkt (UA)	21.07.2017	x	x	x	+	ISH/PCR	ND	ND
Great tit	Graz (St)	24.07.2017	x	emaciated	14	+	ISH/PCR	OL678476	OL654281
Greenfinch	Schwarzenberg (UA)	11.08.2017	M/A	emaciated	18	+	ISH/PCR	OL678477	ND
Greenfinch	Schwarzenberg (UA)	11.08.2017	M/A	emaciated	20	+	ISH/PCR	ND	ND
Greenfinch	Schwarzenberg (UA)	11.08.2017	M/A	emaciated	22	+	ISH/PCR	ND	ND
Great tit	Strasshof (LA)	17.08.2017	F/A	emaciated	12	0	PCR	ND	ND
Greenfinch	Schwarzenberg (UA)	04.09.2017	x	emaciated	22	+	ISH/PCR	ND	ND
Eurasian Siskin	Vienna	15.09.2017	M/A	emaciated	12	+	ISH/PCR	OL678478	OL654282
Yellowhammer	Altenmarkt (St)	07.03.2018	M/A	emaciated	25	+	ISH/PCR	OL678479	OL654283
Greenfinch	Ilz (St)	26.06.2018	M/A	poor	26	+	ISH/PCR	ND	ND
Hawfinch	Hohenau (LA)	05.07.2018	x	emaciated	42	+	ISH/PCR	OL678480	OL654284
Hawfinch	Hohenau (LA)	05.07.2018	x	emaciated	36	+	ISH/PCR	OL678481	OL654285
Greenfinch	Althofen (C)	06.07.2018	M/A	emaciated	18	+	ISH/PCR	OL678482	OL654286
Greenfinch	Schattendorf (B)	10.07.2018	x	emaciated	20	+	ISH/PCR	OL678483	OL654287
Greenfinch	Vienna	31.07.2018	x	emaciated	20	+	ISH/PCR	ND	ND
Greenfinch	Kirchberg (UA)	07.08.2018	x	emaciated	22	+	ISH/PCR	OL678484	ND
Greenfinch	Lanzenkirchen (LA)	16.08.2018	M/A	emaciated	18	+	ISH/PCR	ND	ND
Goldfinch	Lanzenkirchen (LA)	16.08.2018	M/A	good	16	+	ISH/PCR	OL678485	OL654288
Greenfinch	Götzendorf (LA)	16.08.2018	M/A	emaciated	20	+	ISH/PCR	ND	ND
Greenfinch	Loosdorf (LA)	16.08.2018	M/A	emaciated	24	+	ISH/PCR	ND	ND
Greenfinch	Loosdorf (LA)	16.08.2018	M/A	poor	26	+	ISH/PCR	ND	ND
Chaffinch	Loosdorf (LA)	16.08.2018	x	good	24	+	ISH/PCR	OL678486	ND
Chaffinch	Loosdorf (LA)	16.08.2018	x	poor	18	+	ISH/PCR	OL678487	OL654289
Goldfinch	Vösendorf (LA)	22.08.2018	M/A	emaciated	12	+	ISH/PCR	OL678488	OL654290
Greenfinch	Hart b. Graz (St)	23.08.2018	x	emaciated	16	+	ISH/PCR	OL678489	OL654291
Chaffinch	Röthis (V)	29.09.2018	M/A	good	22	+	ISH/PCR	OL678490	OL654291

Species	Location	Date found	Sex/ Age	Body Condition	Weight (g)	Crop lesions	Detection method	GenBank accession numbers ITS1-5.8S-ITS2	Fe-Hydrogenase-gene
Greenfinch	Maria Alm (S)	25.01.2019	M/A	emaciated	18	+	ISH/PCR	OL678491	OL654292
Yellowhammer	Yspertal (LA)	01.02.2019	M/A	emaciated	20	+	ISH/PCR	OL678492	OL654293
Greenfinch	Loosdorf (LA)	05.02.2019	M/A	emaciated	10	+	ISH/PCR	OL678493	OL654294
Chaffinch	Güssing (B)	13.03.2019	M/A	good	22	+	ISH/PCR	OL678494	OL654295
Greenfinch	Kleinbaumgarten (LA)	23.07.2019	M/A	emaciated	18	+	ISH/PCR	ND	ND
Greenfinch	Bergheim (S)	31.07.2019	x	emaciated	16	+	ISH/PCR	OL678495	OL654296
Greenfinch	Wallern (B)	13.08.2019	x	emaciated	14	+	ISH/PCR	OL678496	ND
Greenfinch	Wallern (B)	13.08.2019	x	emaciated	14	+	ISH/PCR	ND	ND
Greenfinch	Wallern (B)	13.08.2019	x	emaciated	18	+	ISH/PCR	ND	ND
Chaffinch	Steyr (UA)	23.03.2019	x	emaciated	15	+	ISH/PCR	ND	ND
Greenfinch	Dornbirn (V)	15.10.2019	M/A	emaciated	19	+	ISH/PCR	OL678497	OL654297
Greenfinch	Dornbirn (V)	15.10.2019	M/A	emaciated	24	+	ISH/PCR	ND	ND
Goldfinch	Loosdorf (LA)	24.01.2020	x	emaciated	13	+	ISH/PCR	OL678498	OL654298
Brambling	Loosdorf (LA)	24.01.2020	x	emaciated	16	+	ISH/PCR	OL678499	OL654299
Eurasian Siskin	Herzogsdorf (UA)	28.01.2020	M/A	emaciated	11	+	ISH/PCR	OL678500	OL654300
Chaffinch	St. Andrä-Höch (St)	13.02.2020	x	good	20	+	ISH/PCR	OL678501	ND
Brambling	St. Andrä-Höch (St)	13.02.2020	x	emaciated	15	+	ISH/PCR	OL678502	ND
Goldfinch	Nappersdorf (LA)	13.03.2020	M/A	emaciated	13	+	ISH/PCR	ND	ND
Bullfinch	Bad Gastein (S)	26.06.2020	M/A	good	25	+	ISH/PCR	ND	ND
Goldfinch	Leopoldsdorf (LA)	03.07.2020	M/A	emaciated	14	+	ISH/PCR	OL678503	OL654301
Greenfinch	Leopoldsdorf (LA)	03.07.2020	F/A	emaciated	19	+	ISH/PCR	OL678504	OL654302
Greenfinch	Manhartsbrunn (LA)	06.07.2020	M/A	emaciated	20	+	ISH/PCR	ND	ND
Bullfinch	Aurach b. Kitzbühel (T)	04.08.2020	x	emaciated	18	+	ISH/PCR	OL678505	OL654303
Hawfinch	Grub (LA)	17.08.2020	F/A	emaciated	39	+	ISH/PCR	OL678506	OL654304
Great tit	Mischendorf (B)	27.08.2020	x	x	x	+	ISH/PCR	OL678507	ND
Eurasian Siskin	Dornbirn (V)	28.09.2020	x	emaciated	10	0	PCR	ND	ND

UA = Upper Austria; St = Styria; B = Burgenland; LA = Lower Austria; V = Vorarlberg; C = Carinthia; S = Salzburg; T = Tyrol; F = female; M = male; A = adult; x = not recorded; g = gram; + = positive; 0 = no changes; ISH = *in situ* hybridization; PCR = polymerase chain reaction; ND = not done.
